# Entire Absence of All the Teeth

**Published:** 1855-04

**Authors:** 


### ARTICLE XII.

### *Entire Absence of all the Teeth.*

One of the most remarkable anomalies has been described to us by a friend, who informs us of his intimate acquaintance with a whole family who have not nor ever had any teeth, although they are all grown and some have families.

The ladies are said to be exceedingly beautiful, and their rosy lips conceal this strange defect. No difficulty is experienced in the mastication of their food, their gums having become hard, even sufficient for the cracking of many kinds of nuts. So much is this the case as to make them decline all the persuasions of their friends to wear artificial teeth.

In fact, they congratulate themselves exceedingly upon their exemption from their liability to the inconvenience of pain and trouble their friends endure from the diseases consequent upon natural teeth. As far as could be learned, they were well and muscularly formed, the men even athletic.

We are somewhat at a loss to account for this strange phenomenon other than as an isolated fact, as one of the freaks of nature. Could a closer inspection be had, much light might be thrown upon this matter. There may be some marked peculiarity in the skin of these individuals that would show some peculiar character, leading to the true cause—either its anatomical structure or its functions of sensibility, secretion or absorption. This would most likely indicate a difference in the mucous membrane, from which the teeth originate, as it is conceded that this is a continuation of the skin. Now we know, that the skin is, at times, wanting in the usual amount of circulating tissue, as also of its secretory and absorption apparel. Under this view we may suggest this the cause, for of course, let the mucous membrane be deficient in circulation, teeth could not be formed.

